# Cost‐Utility Analysis of Routine Anxiety and Depression Screening in Patients Consulting for Osteoarthritis: Results From a Clinical, Randomized Controlled Trial

**DOI:** 10.1002/acr.23568

**Published:** 2018-11-28

**Authors:** Jesse Kigozi, Sue Jowett, Barbara I. Nicholl, Martyn Lewis, Bernadette Bartlam, Daniel Green, John Belcher, Kris Clarkson, Zoe Lingard, Christopher Pope, Carolyn A. Chew‐Graham, Peter Croft, Elaine M. Hay, George Peat, Christian D. Mallen

**Affiliations:** ^1^ Keele University, Keele and University of Birmingham Edgbaston, Birmingham UK; ^2^ University of Glasgow Glasgow UK; ^3^ Keele University Keele UK

## Abstract

**Objective:**

To investigate the cost‐effectiveness (cost‐utility) of introducing general practitioner screening for anxiety and depression in patients consulting for osteoarthritis (OA).

**Methods:**

A cluster‐randomized trial‐based economic evaluation to assess general practitioners screening for anxiety and depression symptoms in patients consulting for OA compared to usual care (screening for pain intensity) was undertaken over a 12‐month period from a UK National Health Service and societal perspective. Patient‐level mean costs and mean quality‐adjusted life years (QALYs) were estimated, and cost‐effectiveness acceptability curves controlling for cluster‐level data were constructed. The base‐case analysis used the net benefit regressions approach. The 2‐stage nonparametric sampling technique was explored in a sensitivity analysis.

**Results:**

The base‐case analysis demonstrated that the intervention was as costly as, and less effective than, the control (QALY differential −0.029 [95% confidence interval −0.062, 0.003]). In the base‐case analyses, general practitioner screening for anxiety and depression was unlikely to be a cost‐effective option (probability <5% at £20,000/QALY). Similar results were observed in all sensitivity analyses.

**Conclusion:**

Prompting general practitioners to routinely screen and manage comorbid anxiety and depression in patients presenting with OA is unlikely to be cost‐effective. Further research is needed to explore clinically effective and cost‐effective models of managing anxiety and depression in patients presenting with clinical OA.

## Introduction

Osteoarthritis (OA) is a common cause of persistent pain, disability, and poor quality of life in older adults. Estimations are that approximately 1 million adults in the UK consult for OA each year 1. OA has negatively impacted the economy of the UK, with direct and indirect costs between 0.25% and 0.50% of gross national product 2, 3. Direct costs of OA treatment are driven mainly by total joint arthroplasty, while indirect costs are mostly driven by the loss of productivity due to absenteeism from paid work 4, 5. Among other factors, persistent pain and disability‐related symptoms have been linked with increased levels of anxiety and depression in patients with OA, who experience subsequent worse outcomes 6, 7. Patients with OA and associated depression use more medication and are likely to have increased health care resource utilization 8, 9. Depression is also a major cause of work‐related absenteeism and diminished or reduced productivity 10.

**Significance & Innovations**

- This is the first economic evaluation assessing general practitioner screening for anxiety and depression in older patients with osteoarthritis (OA) in addition to screening for pain intensity.
- This economic evaluation showed that routine screening for anxiety and depression by general practitioners is unlikely to be cost‐effective in patients with OA.
- Further research is needed to explore cost‐effective models of managing anxiety and depression in patients presenting with clinical OA.

Case finding for depression in primary care has been recommended as a means of improving the identification of depressive symptoms and consequently improving overall quality of life and OA‐related pain outcomes 11. Case finding for depression in patients with a chronic physical disease such as OA has been recommended in guidelines by the National Institute for Health and Care Excellence (NICE) 12, 13, but these guidelines did not consider the costs and benefits of such strategies. There have been calls to investigate the effectiveness and cost‐effectiveness of case finding for anxiety and depression in patients with OA in primary care 14, although to date, evidence of effectiveness and cost‐effectiveness remains limited 15, 16, 17.

We conducted an economic evaluation alongside a randomized controlled trial (RCT) to establish the cost‐effectiveness of introducing general practitioner (GP) screening for anxiety and depression in older patients consulting for OA. The trial and its clinical findings have been reported in full elsewhere 18. Here, we report the cost‐effectiveness (cost‐utility) analysis, giving specific attention to a comparison of alternative methodologic approaches to analyzing cost‐effectiveness data from clinical RCTs.

## Materials and Methods

### Study overview

The economic evaluation took the form of a cost‐utility analysis alongside a clinical RCT over a 12‐month follow‐up period. Randomization was conducted at the GP practice level to prevent contamination between the 2 arms and participating GPs 19. The NHS perspective was adopted in the base‐case analysis. Ethical approval for this study was obtained from the Black Country Research Ethics Committee (#11/WM/0093), and all participants gave informed consent to participate in the study. Primary care resource data were obtained from reviewing medical records, while secondary care resource use data were collected via structured patient questionnaires at baseline and at 2 follow‐up points, 6 months and 12 months. Because the length of follow‐up was 12 months, neither costs nor outcomes were discounted. The primary outcome for clinical effectiveness was patient‐reported current pain intensity on a 0–10 numerical rating scale 20, and the primary time point was during 12 months after consultation. Full details of the trial methods and results have been previously published 18.

### Interventions

Patients in GP practices implementing the intervention were screened by GPs at point‐of‐care for anxiety, depression, and current pain intensity with the aid of an electronic template linked with patient records, which appeared following entry of a Read code for an OA‐related condition. The template incorporated a 2‐item brief depression tool (the Patient Health Questionnaire) 21, and a 2‐item ultrabrief anxiety assessment tool (the Generalized Anxiety Disorder instrument) 22. Negative responses to the anxiety and depression questions were used to rule out a potential depression or anxiety diagnosis. The template then prompted GPs to follow NICE clinical guidelines on the management of OA, anxiety, and depression in patients with physical health problems 23, 24, 25. The control involved point‐of‐care current pain intensity assessment prompted by the electronic template, but involving only the item on current pain intensity. We calculated participant‐specific costs for the intervention based on information collected within the trial. This calculation was made by asking participating GPs about any additional time they spent screening for anxiety and depression. Intervention costs were then estimated, based on an average 1.29 additional minutes of GP time for screening participants and based on a mean of the summary of responses from the participating GPs in the GP questionnaire survey.

### Outcome measures

We used the EuroQol 5‐domain instrument 5‐level version (EQ‐5D‐5L), with a value established using the UK value set derived from a UK general population survey 26, 27 to estimate quality‐adjusted life years (QALYs) gained using the area under the curve approach 28. Imbalances in baseline EQ‐5D‐5L utility scores were controlled for using a multiple regression–based adjustment 29. This approach allows for estimation of differential QALYs and facilitates prediction of adjusted QALYs, while controlling for baseline utility values. The model included the treatment arm dummy variable and patient‐specific baseline utility values 29. The primary outcome for the trial was patient‐reported current pain intensity on a 0–10 numerical rating scale 19 across 12 months after consultation. All outcomes were measured at baseline, 3 months, 6 months, and 12 months and were obtained from a self‐completed questionnaire administered at these time points.

### Resource use and costs

In the base‐case analysis, costs were measured from the UK NHS perspective, with overall societal costs considered in the sensitivity analysis. Information about resource use related to OA, anxiety, and depression was collected from general practice records and self‐reported questionnaires. Primary care resource use was obtained from a review of medical records covering the full 12‐month follow‐up period for patients who provided consent, and included primary care contacts and prescribed medications. Patient‐level data on primary care consultations (from GP records) at which OA, anxiety, and/or depression were mentioned were noted and recorded by type of consultation and type of professional seen. Secondary care resource use data were collected via self‐report postal questionnaires administered at 6 months and 12 months. Secondary care contacts included visits to other health care professionals (e.g., hospital consultants, physiotherapists, counselors, and psychologists), hospital‐based investigations (e.g., radiographs, magnetic resonance imaging scans), and procedures (injections, surgeries). Non‐NHS health care costs were assessed by obtaining information on a patient's purchase of over‐the‐counter medicines, treatments, or appliances and their use of private health care using postal self‐report questionnaires. Self‐reported data on time off from work and occupation were also collected to assess broader economic consequences. Table 1 shows the unit costs and sources used to value health care resources. Unit costs were obtained from the published British National Formulary 30 for estimating the cost of prescribed medication, and from the NHS reference costs 31 and Unit Costs of Health and Social Care 32 for primary and hospital‐based resource use items. Productivity costs were estimated using the human capital approach, and salary costs were based on mean weekly earnings by age and sex and UK Standard Occupational Classification coding 33, 34, 35. All costs were expressed in 2013/2014 UK prices.

**Table 1:** Unit costs and data sources^a^

Health care resource	Unit cost, £
Primary care^b^	
GP consultation per 11.7 minutes	34
Practice nurse consultation per hour	44
Nurse home visit per hour	60
Community physiotherapist per hour	30
Secondary care contacts^c^	
Orthopedic surgeon	128
Rheumatologist	202
Massage therapist	49
Physiotherapist	49
Osteopathic care provider	49
Mental health nurse	34
Chiropractor	49
Psychiatrist	283
Psychologist	264
Intervention cost	
Extra time to complete prompt: 1.29 minutes	2.91
Prescribed medication	Patient‐specific^d^
Medical investigations/interventions	Patient‐specific^c^
Time off work	Mean national wage by age and sex^e^

a Available at: http://www.ons.gov.uk. GP = general practitioner.

b Unit Cost of Health and Social Care 2013 (Personal Social Services Research Unit).

c National Health Service reference costs schedule 2012/2013.

d British National Formulary (2013).

e Annual Survey of Hours and Earnings.

### Statistical analysis

We explored the amount of each resource used by patients in each group using frequencies and mean ± SDs. The statistical analysis was conducted on an intent‐to‐treat basis and in accordance with current RCT guidelines 36. The analysis involved adopting multilevel modeling statistical techniques, taking into consideration clustering in the cost‐and‐effect data 36, 37. The multiple imputation technique using predictive mean matching was used to impute all missing values for the EQ‐5D‐5L and cost data 38. The imputation model included age, sex, and treatment group, and was based on M = 25 imputed data sets.

Separate generalized equation models, controlling for clustering, were used to estimate the mean incremental costs and QALYs for the intervention relative to the control. Uncertainty was examined by estimating 95% confidence intervals and cost‐effectiveness acceptability curves (CEACs), which link the probability of the GP screening intervention being cost‐effective to a range of potential threshold values (λ) that the health system may be willing to pay per additional QALY gained 36, 39, 40. Dependent variables in the multilevel models included costs, QALYs, and net monetary benefits (NMBs), and model coefficient estimates of differences in these variables were used as part of the incremental analysis. Statistical analysis was performed using Stata software, version 12 41.

### Cost‐effectiveness analytical approach

In the base‐case analysis, CEACs were estimated using an NMB regression approach 42. The NMB method allows costs and outcomes to be considered on the same monetary scale. NMB was defined as λ × (Δ effect_i_) − Δ cost_i_, where Δ effect_i_ = the incremental person‐level outcome associated with the screening intervention, Δ cost_i_ = the additional costs due to screening for anxiety and depression, and λ = willingness to pay per unit of outcome gain. Using the output, we plotted CEACs, showing the likelihood that the screening intervention is cost‐effective given different assumptions about willingness to pay for outcomes. The regression analysis adopted methods reflecting the cluster randomized nature of the trial 36, 37, by using a regression‐based model of net benefits, with general practice as the cluster identifier.

### Sensitivity analysis

Sensitivity analyses were performed to assess uncertainty and robustness of the findings. First, CEACs were estimated by using a 2‐stage nonparametric bootstrapping technique for comparison purposes 40, 43. This approach accounts for clustering between the hierarchical cost‐and‐effect data. The 2‐stage bootstrapping output from Stata reports the cost‐effectiveness probabilities for a range of potential threshold values used to estimate the probability that the intervention was cost‐effective. Second, results were presented from a societal perspective, taking into account productivity costs. Third, a complete‐case analysis was carried out to assess the impact of missing cost and EQ‐5D‐5L data.

## Results

### Overview

Overall, 45 general practices were randomized, 24 to the control group and 20 to the intervention group, with a mean practice list size of 7,397 and 5,850 in the control and intervention groups, respectively. In all, 7,279 patients were identified as eligible, and after appropriate exclusions, 2,042 patients were mailed a postconsultation questionnaire. Individual participants recruited from the intervention and control practices had broadly similar characteristics. A summary of participation rates, by arm and by participant baseline characteristics, is shown in Supplementary Tables 1 and 2, available on the *Arthritis Care & Research* web site at http://onlinelibrary.wiley.com/doi/10.1002/acr.23568/abstract. A total of 1,412 participants returned questionnaires from 20 intervention practices (n = 501; 35%) and from 24 control practices (n = 911; 65%) and were considered for the base‐case analysis. Complete primary care resource data from GP records were available for 1,235 participants (87%), while complete secondary care resource data were available for 985 participants (70%) who returned questionnaires at both 6 and 12 months. Complete QALY data at baseline, 6 months, and 12 months were available for 573 intervention participants (63%) and 307 control participants (61%). Following the multiple imputation procedure, all 1,412 participants were included in the base‐case analysis. The mean age of participants was 65 years, with slightly more women (57%), who were overwhelmingly white (97%), with just over 30% in paid employment.

### Resource use

Table 2 shows the disaggregated details of mean resource use for participants with complete resource use data. Service use was summarized under broad categories, and individual items of service use were not imputed. There were minimal differences between the 2 groups in terms of health care use, but the number of GP visits for depression, anxiety, and OA was slightly higher among intervention participants (2.86 versus 2.42), as were visits to other NHS professionals for any reason (0.26 versus 0.13). Similar findings were observed when considering a societal perspective, with the exception of significantly higher reported time off from work in the control group at 12 months (15 days versus 8.3 days).

**Table 2:** Health care resource use and costs by trial arm^a^

	Resource use, units		Cost, £	
Resource/cost component	Control (n = 633)	Intervention (n = 352)	Control (n = 633)	Intervention (n = 352)
Primary care^b^				
General practitioner	2.42 ± 2.07^c^	2.86 ± 2.25^c^	80.92 ± 68.19	94.38 ± 73.23
Practice nurse	0.03 ± 0.18	0.03 ± 0.17	0.23 ± 1.57	0.27 ± 1.55
Other	0.07 ± 0.34	0.04 ± 0.20	–	0.14 ± 1.67
Secondary care appointments^d^				
NHS consultant	2.07 ± 4.38	1.69 ± 2.94	134.93 ± 262.94	112.45 ± 207.82
Private consultant	0.44 ± 1.93	0.41 ± 1.70	23.48 ± 104.19	21.13 ± 80.89
Other professionals, NHS hospitals	0.13 ± 0.53^c^	0.26 ± 1.24^c^	10.58 ± 36.41	17.48 ± 48.47
Other professionals, private hospitals	0.06 ± 0.56	0.05 ± 0.50	0.07 ± 1.74	0.25 ± 3.31
Investigations and treatments^d^	372 (58.8)^e^	197 (55.9)^e^	377.21 ± 1,153.01	353.33 ± 1,014.55
Prescriptions				
Anti‐anxiety/anti‐depression drugs, no. (%)	150 (19)	100 (23)	–	–
OA drugs, no. (%)	567 (71)	343 (78)	–	–
Prescribed medication	–	–	225.49 ± 205.80	225.59 ± 190.22
Over‐the‐counter medicines/treatments^d^	373 (58.9)^e^	201 (57.10)^e^	13.55 ± 50.29	14.10 ± 58.46
Productivity loss/costs^f^	15.01 ± 31.96	8.34 ± 16.95	1,094.87 ± 2,530.32	644.25 ± 1,460.85
Productivity costs^g^	–	–	310.74 ± 1,441.57	174.59 ± 816.72

a Values are the mean ± SD per patient, by treatment group, for patients providing health care utilization data, unless indicated otherwise.

b Data based on medical records review of available data and include anxiety/depression and OA‐related health care use.

c Significant differences between the groups, because the value zero is not contained in the 95% confidence interval. Confidence intervals for mean differences in resource use were obtained by bias‐corrected and accelerated nonparametric bootstrapping, using 1,000 replications.

d Data based on self‐reported questionnaires at 6 and 12 months and include secondary health care use for any health reason.

e Number (%) of participants reporting usage are given instead of mean ± SD because of multiple usage, purchases, and/or prescriptions over 12 months.

f Indirect costs based on the subsample of respondents in paid employment at 12 months (n = 280).

g Indirect costs based on the complete‐case data set (n = 985).

### Health care costs and productivity costs

Table 2 summarizes the mean cost per patient, by group, for each category of cost. All available data are included for each category. Comparing the 2 groups, we found small differences in the costs, with the exception of significant differences in costs from other health care professionals (such as occupational therapists, acupuncturists, and chiropractors) and from productivity loss. NHS, health care, and societal costs were similar in both groups, although slightly higher in the control arm. Adjusting for clustering in costs resulted in negligible differences between the groups.

At 12 months of follow‐up, 98 in the intervention group (26.4%) and 182 in the control group (28.2%) were in paid employment. Of those reporting being employed, 75 patients (11.6%) in the intervention group reported time off from paid work compared with 35 (9.4%) in the control group. Over a period of 12 months of follow‐up, the mean days taken off from work was higher in the control arm (15.0 days) than the intervention arm (8.3 days), and this difference translated to higher productivity costs in the control arm compared to the intervention arm (Table 2).

### Health outcomes and estimation of cost‐effectiveness

The results for analysis of the health outcome measures (mean EQ‐5D‐5L scores and QALYs) are shown in Table 3. In terms of QALYs gained at 12 months, the mean estimates were 0.686 for the screening intervention and 0.711 for the control, showing higher QALY scores for the control group after adjusting for baseline differences. A similar result was observed in the unadjusted QALY scores.

**Table 3:** Cost‐utility analysis using the net benefit regression approach^a^

	Control (n = 911)	Intervention (n = 501)	Incremental analysis, intervention vs. control, mean differences (95% CI)^b^
Cost analysis, £			
NHS cost	759.74 ± 1152.38	744.09 ± 1008.36	1.02 (−135.96, 138.02)
Health care cost	794.73 ± 1163.13	777.75 ± 1031.82	2.70 (−138.28, 143.69)
Societal cost	1071.69 ± 1866.80	946.34 ± 1351.82	−122.29 (−318.50, 73.91)
Effectiveness analysis			
Unadjusted QALYs gained	0.715 ± 0.216	0.679 ± 0.220	−0.029 (−0.062, 0.003)^c^
Adjusted QALYs gained	0.711	0.686	–

a Values are the mean ± SD unless indicated otherwise. Data set is imputed. 95% CI = 95% confidence interval; QALY = quality‐adjusted life year.

b Mean differences adjusted for clustering in cost and QALY outcomes.

c *P* = 0.072.

### Estimation of cost effectiveness

**Figure 1:**
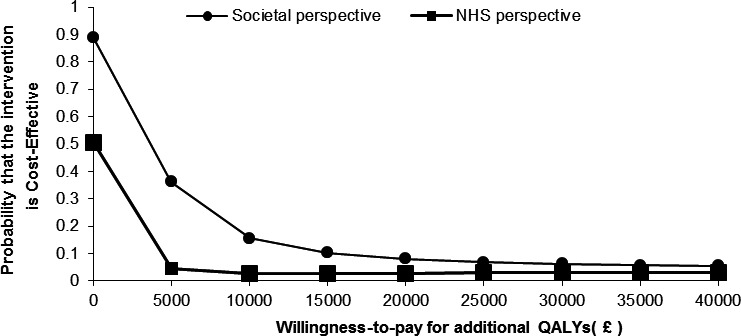
Cost‐effectiveness acceptability curve for the comparison of case finding versus pain only based on the net monetary benefit regression approach. QALYs = quality‐adjusted life years.

Estimates showed that the intervention was associated with lower QALYs and that the adjusted difference in cost between the 2 groups was minimal (£1.02 lower in the intervention arm). The mean cost and outcome results of the cost‐utility analysis based on the net monetary benefit approach and forming the base‐case analysis are shown in Tables 3 and 4. The findings indicate that there is a very low probability (<10%) of the intervention being cost‐effective at conventional willingness‐to‐pay thresholds for additional QALYs (£20,000 to £30,000 per QALY). Figure 1 shows the probability that the screening intervention would be seen as cost‐effective using a CEAC for different values of willingness to pay from an NHS and societal perspective–based net monetary benefit approach.

**Table 4:** Cost‐effectiveness analysis using the net benefit regression approach, with probability that treatment is cost effective at λ^a^

Threshold value estimate	NHS	Societal
λ = £0	51	89
λ = £5,000	4	36
λ = £10,000	3	16
λ = £15,000	3	10
λ = £20,000	3	8
λ = £25,000	3	7
λ = £30,000	3	6
λ = £35,000	3	6
λ = £40,000	3	5

a Values are the percentage. Data set is imputed. λ = willingness to pay per unit of outcome gain.

### Sensitivity analysis results

**Figure 2:**
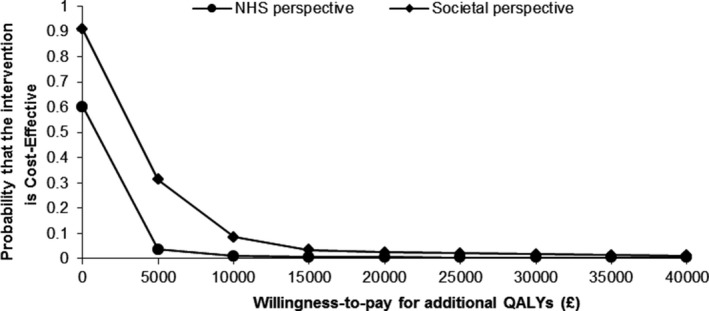
Cost‐effectiveness acceptability curve for the comparison of case finding versus pain only based on the 2‐stage bootstrap sampling process. QALYs = quality‐adjusted life years.

Similar results were observed when using the 2‐stage nonparametric bootstrap approach. The probability of the intervention being cost‐effective remained low (<10%) at a conventional willingness‐to‐pay threshold for additional QALYs. The output results from this approach were, however, slightly lower than the net monetary benefit approach (Figure 2). The analysis conducted from a societal perspective showed that results were broadly similar to the findings in the base‐case analysis (Figures 1 and 2). The results from the complete‐case sensitivity analysis are shown in Supplementary Table 3 and in Supplementary Figures 1 and 2, available on the *Arthritis Care & Research* web site at http://onlinelibrary.wiley.com/doi/10.1002/acr.23568/abstract. The results from these analyses were generally consistent with the findings from the base‐case analysis, with the intervention as costly as but less effective than the control. The only exception was that the intervention was associated with a slightly higher probability of being cost‐effective (20%) in both approaches when compared to the base‐case analysis.

## Discussion

This study reports a cost‐utility analysis of introducing GP screening for anxiety and depression in older patients consulting for OA, and an assessment of alternative analytical approaches for economic evaluation of clinical RCTs. The results showed that GP screening for anxiety and depression in OA patients is unlikely to be a cost‐effective option, and similar results are observed regardless of the perspective or approach adopted for the economic analysis. There was minimal difference between the 2 groups in terms of costs, and the finding that additional screening for anxiety and depression is unlikely to be cost‐effective is primarily based on the evidence that shows slightly higher QALYs in the control group. The results supplement the main trial findings that found a small significant difference in the direction of worse pain outcomes among those screened for depression and anxiety compared to those not screened 18. Further exploration of these results using probability bias analysis suggests the results could plausibly be attributed to selection bias 44. In both analytical approaches considered, probability of GP screening for anxiety and depression being cost‐effective remained low (<30%) at the recommended NICE thresholds of £20,000 to £30,000 45.

This is the first economic evaluation assessing GP screening for anxiety and depression in older people with OA in addition to screening for pain intensity. The study was based on a large sample of a wide range of patients with clinically diagnosed OA (n = 1,412) across a number of locations (45 practices). Resource use information was collected using a combination of GP records and self‐reported data, including information outside the main NHS perspective, and therefore the study reports comprehensive resource use data. However, a limitation of only using self‐report data is that respondents could potentially underreport resource utilization, particularly over longer periods of recall 46, 47. A further strength is that the use of the QALY measure in the cost‐effectiveness analysis may have captured a broader range of effects of screening than the pain measure used in the clinical article, given that the EQ‐5D‐5L used to generate the QALY measure includes dimensions of pain, mobility, anxiety, and depression. In addition, the analysis used a pragmatic and rigorous study design and employed comparative statistical methods for analyzing cost‐effectiveness data alongside cluster trials, based on recommended methods 36, 48.

However, there are also some limitations. The amount of missing data in the primary care and secondary care data requiring imputation may be of concern. Response rates for the secondary care costs (70% at 12 months) and QALYs (62% over 12 months) were relatively low. Multiple imputation using predictive mean matching was used to address potential biases resulting from incomplete data. However, a limitation of this approach is that it does not take into consideration the multilevel structure of the data. Moreover, the imputation model specified could have included a wider set of variables and covariates. Results of the base‐case (imputed) and complete‐case analysis were comparable in regard to policy implications.

Evidence supporting the effectiveness of screening for anxiety and depression in patients with OA remains limited and with contradictory findings 14, 18, 49. No previous studies have assessed the cost‐effectiveness of GP screening for anxiety and depression in addition to screening for pain intensity in patients with OA at risk of depression. Our study, in line with findings from a previous review 16, suggests that screening for anxiety and depression in general is unlikely to be a cost‐effective option.

Few studies have attempted to compare alternative methods for the economic analysis of clinical RCTs 48, although methodologic guidance on the use of these methods to analyze data from clinical RCTs has been established. The analysis reported in this article showed similar findings from the different approaches explored (net monetary benefit approach and 2‐stage bootstrap sampling process). The results obtained here may not be generalizable to another disease or context. Further empirical studies exploring alternative methods of analyzing clinical RCTs in other disease areas or contexts are needed.

Our results show that patients receiving a screening intervention for depression, anxiety, and pain intensity were associated with higher primary care costs, but lower hospital and productivity loss costs. However, patients receiving the screening intervention were associated with slightly lower quality of life outcomes. The economic evaluation demonstrated that adding routine screening for anxiety and depression compared to usual care is not a cost‐effective option for patients with OA. The study has broader implications in depression case finding interventions targeted at patients with OA, particularly with the assumption that such services are money‐saving to the NHS and improve overall OA‐related health outcomes. Here, productivity and hospital cost savings were observed, but the probability of overall cost‐effectiveness was judged very low at recommended incremental cost‐effectiveness ratio thresholds.

Nonetheless, given the significant costs associated with OA and the increased risks of anxiety and depression in this group of patients, future research should explore the costs and benefits of appropriate management strategies for anxiety and/or depression once detected in patients presenting with clinical OA in primary care. Assessing ways of identifying those anxiety or depressive symptoms that are likely to make future management services both less cost‐effective and more costly would also be helpful.

## Author Contributions

All authors were involved in drafting the article or revising it critically for important intellectual content, and all authors approved the final version to be submitted for publication. Dr. Jowett had full access to all of the data in the study and takes responsibility for the integrity of the data and the accuracy of the data analysis.

### Study conception and design

Nicholl, Bartlam, Green, Belcher, Pope, Chew‐Graham, Croft, Hay, Peat, Mallen.

### Acquisition of data

Kigozi, Jowett, Lewis, Clarkson, Lingard.

### Analysis and interpretation of data

Kigozi, Jowett, Lewis, Mallen.
