# Supplementary material for: Cost‐Utility Analysis of Routine Anxiety and Depression Screening in Patients Consulting for Osteoarthritis: Results From a Clinical, Randomized Controlled Trial
Source: Arthritis Care Res (Hoboken). 2018 Nov 28;70(12):1787–94. doi: 10.1002/acr.23568 (PMC6563477; doi:10.1002/acr.23568)
Supplement: Supplementary file 1 [file ACR-70-1787-s001.docx]

**Supplemental tables**

**S1 Table**: Summary of participation rates, by arm

|  | Control | Intervention |
| --- | --- | --- |
| Number of practices randomised and received intervention | 24 | 20 |
| Practice list size, mean (SD) ^†^ | 7,397 (4,250) | 5,850 (2,693) |
| Total number of potentially eligible patients | 4238 | 3041 |
| Ineligible/excluded/declined/refused | 2899 | 2338 |
| Mailed post-consultation questionnaire | 1339 | 703 |
| Returned post-consultation questionnaire | 911 | 501 |
| Returned follow-up questionnaire at 3 months | 708 | 385 |
| Returned follow-up questionnaire at 6 months | 686 | 378 |
| Returned follow-up questionnaire at 12 months | 646 | 371 |

† Practice allocation was based on a minimisation algorithm, with the average practice list size in the control higher than that of practices in the intervention group.

**S2 Table**: Participant baseline characteristics, by arm

|  | Control | Intervention |
| --- | --- | --- |
| Age (years), mean (SD) | 24 | 20 |
| Female, n (%) | 7,397 (4,250) | 5,850 (2,693) |
| Currently in a paid job, n (%) | 286 (31) | 166 (33) |
| Self-reported body mass index (kg/m2), mean (SD) | 28.7 (5.5) | 28.6 (5.1) |
| Comorbidity†, n (%) | 666 (73) | 370 (74) |
| First pain consultation episode, n (%) | 355 (40) | 163 (33) |
| Pain score at time of consultation (0-10), mean (SD) | 6.3 (2.1) | 6.3 (2.0) |
| †Any of the following self-reported conditions: previous heart attack of stroke, angina, raised blood pressure, diabetes, circulation problems in legs, cancer, liver disease, kidney disease, asthma/bronchitis, deafness, or eyesight problems | | |

**S3 Table:** Cost-utility analysis using the net benefit-regression approach

| **COST ANALYSIS** | **Control** | **Intervention** |  |
| --- | --- | --- | --- |
|  | **N=633** | **N=352** |  |
| NHS cost (£)  Mean (Standard Deviation) | 795.45 (1291.72) | 770.40 (1112.73) | |
|  | Incremental Analysis  (Intervention versus Control) | | |
| Adjusted NHS Cost (£)  Difference in Means (95% CI’s) | -23.05 (-189.22 to 143.14) | | |
| **EFFECTIVENESS ANALYSIS** | **Control** | **Intervention** |  |
|  | N=573 | N=307 |  |
|  |  |  | |
| EQ-5D scores (complete-cases) |  |  | |
| Baseline | 0.5888 (0.2440) | 0.5757 (0.2277) | |
| Month 3 | 0.6125 (0.2369) | 0.5831 (0.2366) | |
| Month 6 | 0.6252 (0.2459) | 0.5901 (0.2435) | |
| Month 12 | 0.6338 (0.2450) | 0.6128 (0.2465) | |
| QALYs (complete cases) |  |  | |
| Unadjusted QALYs gained  Mean (Standard Deviation) | 0.7223 (0.214) | 0.7042 (0.222) | |
| adjusted QALYs gained | 0.7224 | 0.7040 | |
|  | Incremental Analysis  (Intervention versus Control) | | |
| QALYs gained  Difference in Means* (95% CI’s) [p-value] | -0.014 (-0.053 to 0.025) [p=0.482] | | |
| **COST EFFECTIVENESS ANALYSIS** | Probability that Treatment is Cost Effective at λ | | |
| Threshold Value (λ)  Estimate | NHS | | |
| λ = £0 | 48% | | |
| λ = £5,000 | 24% | | |
| λ = £10,000 | 22% | | |
| λ = £15,000 | 22% | | |
| λ = £20,000 | 22% | | |
| λ = £25,000 | 22% | | |
| λ = £30,000 | 23% | | |
| λ = £35,000 | 23% | | |
| λ = £40,000 | 23% | | |

NHS, National Health Service, QALY, Quality-Adjusted Life Year. * Mean differences adjusted for clustering in cost and QALY outcomes.

**Supplementary figures**


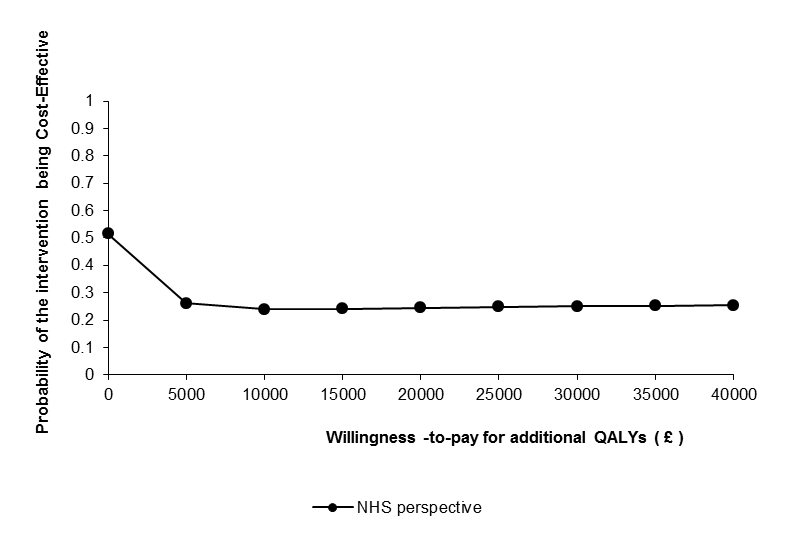


**S1 Figure:** Cost-effectiveness acceptability curve for the comparison of case-finding versus pain only from an NHS perspective based (based on the complete-case dataset)

**S2 Figure:** Cost-effectiveness acceptability curve for the comparison of case-finding versus pain only from an NHS perspective based on the two-stage bootstrap approach (complete-case dataset)
